# Treatment delays in patients with lung cancer: a retrospective study conducted at the National Cancer Institute of Mexico between 2004 and 2021

**DOI:** 10.1093/oncolo/oyae358

**Published:** 2025-04-09

**Authors:** Elysse Bautista-González, Teresa Verenice Muñoz Rocha, Enrique Soto-Perez-de-Celis, Cecilia Vindrola-Padros, Anne Peasey, Hynek Pikhart

**Affiliations:** Institute of Epidemiology and Health Care, Research Department of Epidemiology and Public Health, University College London, London, WC1E 7HB, United Kingdom; Center for Research in Nutrition and Health, National Institute of Public Health, Cuernavaca, Morelos, 62100, Mexico; Division of Medical Oncology, University of Colorado Anschutz Medical Campus, Anschutz Cancer Pavilion, 1665 Aurora Ct, Aurora, CO 80045, Estados Unidos; Division of Surgery & Interventional Science, Department of Targeted Intervention, University College London, London, W1W 7TY, United Kingdom; Institute of Epidemiology and Health Care, Research Department of Epidemiology and Public Health, University College London, London, WC1E 7HB, United Kingdom; Institute of Epidemiology and Health Care, Research Department of Epidemiology and Public Health, University College London, London, WC1E 7HB, United Kingdom

**Keywords:** health services research, cancer, México, treatment interval

## Abstract

**Importance:**

Lung cancer management involves navigating a complex pathway from symptom onset to treatment initiation, where delays can compromise outcomes.

**Objective:**

To identify the length of treatment intervals among Mexican lung cancer patients, compare treatment intervals to results from other countries, and identify determinants of delays.

**Design:**

Retrospective study collecting patient records and exploring the treatment interval in lung cancer.

**Setting:**

The study was conducted at Mexico’s National Cancer Institute.

**Participants:**

2645 lung cancer patients with a confirmed diagnosis between 2004 and 2021 were included in the analysis.

**Exposure:**

Social determinants of health.

**Main Outcome:**

Treatment interval (from diagnosis to treatment).

**Results:**

Logistic regression models revealed significant associations between delays and various factors, including marital status, education, region, first symptom at presentation, treatment type, and political period. A comparison with international guidelines highlighted substantial delays in patients diagnosed at the Instituto Nacional de Cancerología and diagnosed externally.

**Conclusions:**

Targeted interventions should consider patient characteristics to enhance care efficiency. Concerns should be raised about the observed increase in treatment intervals from 2014 and the associated impact on survival rates. There is an urgency for timely interventions, continuous research, and collaborative efforts to optimize care delivery and outcomes for lung cancer patients in Mexico.

**Implications for Practice**

- Treatment intervals have increased from 2004 to 2021.
- The pathway to treatment impacts patients’ outcomes. collaborative efforts to homogenize pathways should be prioritized.
- More research needs to be done to understand the pathway to treatment, the patient journey, and the factors that facilitate or become an obstacle for treatment between population groups.

## Background

Managing lung cancer involves several processes that span from the initial presentation of symptoms to diagnostic evaluation, referrals, and the commencement of treatment.^1-3^ Delays in lung cancer diagnosis become missed opportunities for timely follow-up of radiologically detected suspicious lesions.^2,4^ In turn, this increases the risk of death due to accelerated tumor growth and thus reduces the use of effective treatment options.^5-7^ Additionally, delays in diagnosis and treatment may have an effect on overall survival and also increase patient anxiety and distress.^5,8,9^

**Figure 1.**
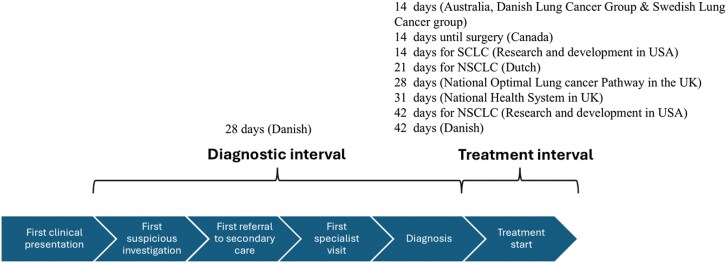
International published guidelines in lung cancer care timeliness: DLCG: Danish Lung Cancer Group; NHS: National Health Service; NOLCP: National Optimal Lung Cancer Pathway in the UK; NSCLC: non-small cell lung cancer; RAND: Research and Development in the USA; SCLC: small cell lung cancer; SLCG: Swedish Lung Cancer Group.^5^

In the literature, findings around time intervals in lung cancer have been inconsistent due to a lack of data and the use of different interval definitions.^5^ Measuring time intervals across cancer care is key to identify where in the patient pathway there is a need for intervention. Time intervals in the pathway to diagnosis and treatment have been examined extensively by researchers.^10,11^ Recommendations from various sources emphasize specific time-frames for key steps in the care process, such as the time between visits with general practitioners and specialist consultations.^5,9^ For instance a 14-day maximum time interval from general practitioner referral to first lung cancer specialist appointment; and 31 days from diagnosis to first treatment.^5,12^ Although recommendations vary, all highlight the importance of minimizing delays.^5,9^Figure 1 summarizes the time intervals recommended by international guidelines for lung cancer care. In Mexico, the lack of research on delays in lung cancer indicates the need for further analysis to optimize patient care and better understand the underlying factors that generate worse outcomes.^13^ Identifying these delays could help develop strategies to improve patient outcomes and healthcare system efficiency.^13^ In this study, we evaluated delays in care for lung cancer in a single Mexican institution and compared them with international frameworks to assess whether they fall within recommendations from the NHS in the United Kingdom (31 days).

## Methods

This study aimed to investigate the determinants of treatment delays among patients with lung cancer who were treated at the National Cancer Institute (Instituto Nacional de Cancerología, INCAN) of Mexico between the years 2004 and 2021. The study included a cohort of 2645 patients with lung cancer. These patients represented a diverse geographic distribution within Mexico. Data for this study were collected directly from electronic health records in 2020-2021. A Redcap extraction sheet was developed to facilitate the systematic retrieval of patient information. Prior to the analysis, data were subjected to a cleaning process. Outliers in the dataset were identified and treated by converting them to missing values. While date variables were automatically generated by the Electronic Health Record software during each consultation, for participants whose dates were not captured automatically, dates were extracted from the clinical record narrative. This approach mirrors a methodology previously employed in a British prospective cohort study.^2^ In instances where the record described symptoms within the last month without specifying the exact date, the mid-month was used as a proxy for the first symptom date. Similarly, for a period specified as “a year,” the mid-year was utilized.^2^ If the clinical record indicated a time frame as “2 weeks ago,” 2 weeks were calculated back from the time the record was filled in to estimate the relevant date. Consequently, multiple dates crucial for this research were identified and collected. The study was conducted following ethical guidelines, and ethical approvals were obtained at University College London and locally at INCAN (020/043/ICI)(CEI/1493/20).

**Figure 2.**
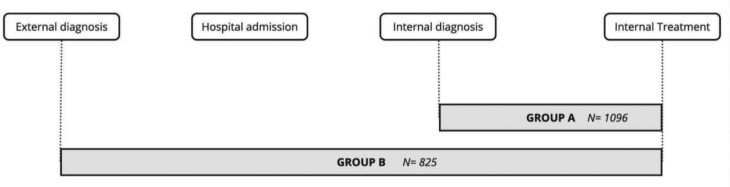
Sample size in Groups A and B and interval definition (*N* = 1096 patients diagnosed internally, and *N* = 825 patients diagnosed before admission).

The primary outcome was defined as the time elapsed from the date of lung cancer diagnosis to the initiation of treatment. After dropping patients with missing diagnosis date, 2 subgroups of population arose: those with a diagnostic suspicion of lung cancer, which was confirmed at INCAN (*N* = 1096) (Group A), and those who presented at INCAN with a confirmed lung cancer diagnosis from an external provider (*N* = 825) (Group B). Figure 2 shows the sample and events that differentiate these 2 groups. The continuous outcome for both groups was categorized as ≤31 days vs >31 days, with the latter serving as the cutoff point to identify health system-related delays as established by the literature.^5^ The date of diagnosis was determined based on the date indicated in the pathology report or the initial date of clinical diagnosis in the medical record (in cases where pathology results were unavailable). “Date-of-external-diagnosis” refers to patients who were diagnosed in another hospital prior to their arrival at INCAN. “ The term “date-of-treatment” was defined as the first instance when a systemic, local, or palliative treatment was administered to the patient.

The study variables’ descriptive statistics were analyzed for groups A and B. A bivariate analysis of participant characteristics was conducted concerning the treatment interval, using chi-square tests to identify differences between groups. Subsequently, a multivariable logistic regression analysis was performed to assess the impact of selected independent variables on the likelihood of experiencing ≤31 days vs >31 days to treatment for each group. The logistic model was chosen for this analysis because of the dichotomous outcome, as it allows for modeling the probability of occurrence of a binary event. Additionally, it provides a clear interpretation through odds ratios, facilitating the understanding of the impact of independent variables on the likelihood of experiencing longer or shorter treatment intervals. The logistic model also does not require independent variables to maintain a linear relationship with the dependent variable, making it more flexible for our data.^14^ This choice aligns with previous studies in the literature that have used the logistic model to analyze delays in medical care,^15^ providing a solid foundation for our findings and demonstrating the robustness of the model.

In the model analysis, an un-adjusted logistic regression was carried out. Later an adjusted logistic regression model was built based on a priori hypotheses and theoretical considerations. The main independent variables were selected based on their relevance to delays in lung cancer care delays found in the literature. Interactions between sex, age, and treatment type were tested within the model, but none were found to be significant. The final models for each group were adjusted for age group, sex, marital status, education, region of origin, primary symptom, type of lung cancer, cancer stages (I-IV), treatment, modalities, and time of admission. Once the model was developed, goodness-of-fit measures were estimated, including the classification of predicted and observed values, showing a concordance of 78.39% for group A’s model and 79.77% for group B’s model. Additionally, the Hosmer-Lemeshow goodness-of-fit statistic was estimated, where a *P*-value of .38 was obtained for model A and a *P*-value of .16 for group B’s model, indicating that our models fit the data well. STATA V17 software was used for data analysis.

## Results

**Figure 3.**
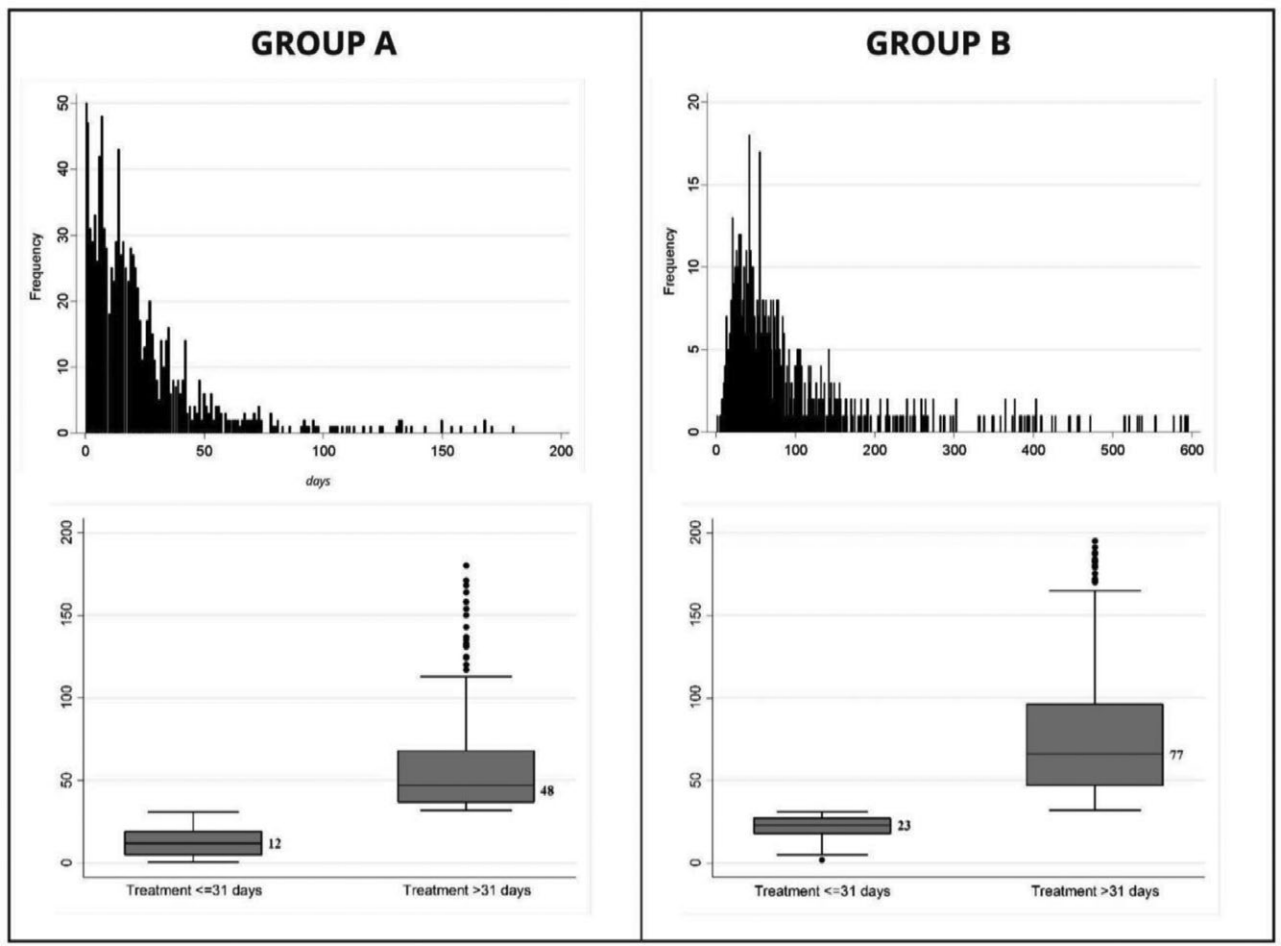
Comparison of Groups A and B continuous and binary distribution of treatment interval.

After arriving at INCAN, a diagnosis of lung cancer was confirmed in 77% of admitted patients, of which 91% received treatment. Patients who received their diagnosis before arriving at INCAN (Group B); were younger, had a higher educational level, and were more likely to present with cough as the initial symptom. Full pathological and radiological information was less likely to be available for patients who underwent their workup before arriving at INCAN, and more likely to end up receiving treatment with TKIs. The time to treatment in both groups shows skewness in the outcomes (Figure 3).

Patients diagnosed by an external provider had a longer wait before starting treatment, with a median time of 62 days, compared to 16 days for patients diagnosed at INCAN. For patients who received their diagnostic workup internally, those without delays (31 days or less) started treatment after a median of 12 days (interquartile range [IQR]: 5.0-19.0 days). In contrast, patients with delays (>31 days) had a median time to treatment of 48.5 days (IQR: 37-73 days). For patients who received their diagnostic workup externally, those without delays (31 days or less) had a median time to treatment of 23 days (IQR: 18.0-27.0 days). For those with delays (>31 days), the median time to treatment was 77 days (IQR: 51-145 days) (Figure 3).

### Factors associated with treatment delays among patients who received their diagnostic workup at INCAN (Group A)

Complete data were available for 801 individuals who received their diagnostic workup for lung cancer at INCAN (Table 1). Widowed individuals, as well as those presenting with dyspnea as the initial symptom, were significantly more likely to experience treatment delays of >31 days. In contrast, patients with elementary school, those diagnosed with small cell lung cancer (SCLC), those receiving radiotherapy as a single treatment modality, and those who received 3 or more treatment modalities were significantly less likely to experience treatment delays of >31 days. Likewise, those diagnosed between 2007 and 2018 were less likely to experience treatment delays of >31 days than those diagnosed between 2019 and 2021.

**Table 1.** Factors related with treatment delays of >31 days among Group A individuals who received their diagnostic workup for lung cancer at INCAN (*N* = 801, *R*^2^ = 12%).

Variables and categories		Odds ratio	*P*-value	CI 95%	
Age group (years)	<49	Reference			
	50-59	1.130	.698	0.611	2.088
	60-69	1.176	.573	0.669	2.066
	≥70	1.011	.970	0.561	1.823
Sex	Men	Reference			
	Women	1.361	.114	0.929	1.992
Marital status	Married	Reference			
	Divorced	1.120	.695	0.635	1.977
	Single	1.407	.249	0.788	2.512
	Widowed	1.945	.013	1.151	3.287
Education	≥High school	Reference			
	Middle school	1.193	.653	0.553	2.573
	Elementary school	0.609	.023	0.396	0.934
	No education	1.013	.970	0.534	1.918
Region of origin	Mexico City	Reference			
	North	1.777	.216	0.715	4.420
	Center	1.487	.097	0.931	2.376
	South	1.516	.129	0.886	2.593
Primary symptom	Cough	Reference			
	Dyspnea	2.299	.002	1.369	3.862
	Chest pain	1.132	.699	0.604	2.120
	Hemoptisis	1.061	.915	0.361	3.119
	Weight loss	1.516	.312	0.677	3.395
	Other symptoms	1.138	.607	0.696	1.861
Type of lung cancer	Non Small Cell Lung Cancer (NSCLC)	Reference			
	Small Cell Lung Cancer (SCLC)	0.103	.034	0.013	0.838
	Unknown/undetermined	0.441	.083	0.175	1.112
Cancer stages I-IV	I and II	Reference			
	III	0.825	.648	0.362	1.883
	IV	1.137	.689	0.607	2.128
	Unknown	0.983	.979	0.264	3.662
Treatment modalities	Single treatment modality (chemo)	Reference			
	Single treatment modality (immunotherapy)	1.607	.753	0.084	30.781
	Single treatment modality (radiotherapy)	0.309	<.0001	0.169	0.562
	Single treatment modality (palliative care only)	0.431	.151	0.137	1.359
	Single treatment modality (tyrosine kinase inhibitor)	0.626	.095	0.362	1.085
	Single treatment modality (other)	4.706	.095	0.764	29.005
	Double treatment modality	1.124	.728	0.582	2.171
	≥Triple treatment modality	0.192	.035	0.042	0.888
	Not specified	0.532	.210	0.199	1.426
Time period of admission	2019-2021	Reference			
	2013-2018	0.455	.020	0.234	0.885
	2007-2012	0.193	<.0001	0.092	0.402
	2004-2006	0.890	.769	0.408	1.940

### Factors associated with treatment delays among patients who received their diagnostic workup before arriving at INCAN (Group B)

Complete data were available for 582 individuals who received their diagnostic workup for lung cancer before arriving at INCAN (Table 2). Patients residing in the northern region of the country, those presenting with weight loss as the initial symptom, those treated with best supportive care only as the single treatment modality and those who received 3 or more treatment modalities were less likely to experience treatment delays of >31 days. Patients diagnosed in the years 2007-2012 were less likely to experience treatment delays of >31 days compared to those diagnosed between 2019 and 2021.

**Table 2.** Factors related with treatment delays of >31 days among Group B individuals who received their diagnostic workup for lung cancer before arriving at INCAN (*N* = 582, *R*^2^ = 9%).

Variables and categories		Odds ratio	*P*-value	CI 95%	
Age group (years)	<49	Reference			
	50-59	0.558	.075	0.293	1.062
	60-69	0.755	.390	0.398	1.433
	≥70	0.725	.360	0.364	1.445
Sex	Men	Reference			
	Women	0.944	.799	0.604	1.474
Marital status	Married				
	Divorced	1.408	.386	0.650	3.050
	Single	1.243	.520	0.641	2.409
	Widowed	1.283	.536	0.583	2.824
Education	≥High school	Reference			
	Middle school	1.417	.456	0.566	3.546
	Elementary school	1.621	.082	0.941	2.791
	No education	1.383	.509	0.528	3.622
Region of origin	Mexico City	Reference			
	North	0.325	.010	0.138	0.765
	Center	0.862	.614	0.483	1.536
	South	0.705	.289	0.369	1.347
Primary symptom	Cough	Reference			
	Dyspnea	1.137	.717	0.569	2.270
	Chest pain	1.231	.582	0.588	2.580
	Hemoptisis	0.529	.385	0.125	2.230
	Weight loss	0.241	.003	0.093	0.626
	Other symptoms	0.774	.389	0.432	1.387
Type of lung cancer	Non Small Cell Lung Cancer (NSCLC)	Reference			
	Small Cell Lung Cancer (SCLC)	0.531	.348	0.142	1.991
	Unknown/undetermined	0.657	.277	0.308	1.402
Cancer stage I-IV	I and II	Reference			
	III	1.072	.925	0.250	4.602
	IV	1.082	.901	0.310	3.779
	Unknown	1.018	.980	0.242	4.281
Treatment type	Single treatment modality (chemo)	Reference			
	Single treatment modality (immunotherapy)	1.000			
	Single treatment modality (radiotherapy)	0.968	.938	0.422	2.221
	Single treatment modality (palliative care only)	0.391	.025	0.172	0.890
	Single treatment modality (tyrosine kinase inhibitor)	1.093	.807	0.536	2.228
	Single treatment modality (other)	0.276	.122	0.054	1.411
	Double treatment modality	0.770	.413	0.412	1.440
	≥Triple treatment modality	0.378	.035	0.153	0.936
	Not specified	0.766	.753	0.146	4.026
Time period of admission	2019-2021	Reference			
	2013-2018	0.662	.227	0.339	1.293
	2007-2012	0.371	.008	0.179	0.768
	2004-2006	0.771	.680	0.225	2.647

## DISCUSSION

The results of our study show that Mexican patients with lung cancer exhibit significant delays in treatment initiation, and that such delays are closely related to the site in which their initial diagnostic workup takes place. Interestingly, patients who underwent a diagnostic workup and had some form of diagnostic confirmation before arriving at the treatment facility (INCAN) had a longer time to treatment initiation than those who underwent their diagnostic workup at INCAN (with a median difference between groups of 46 days).

**Figure 4.**
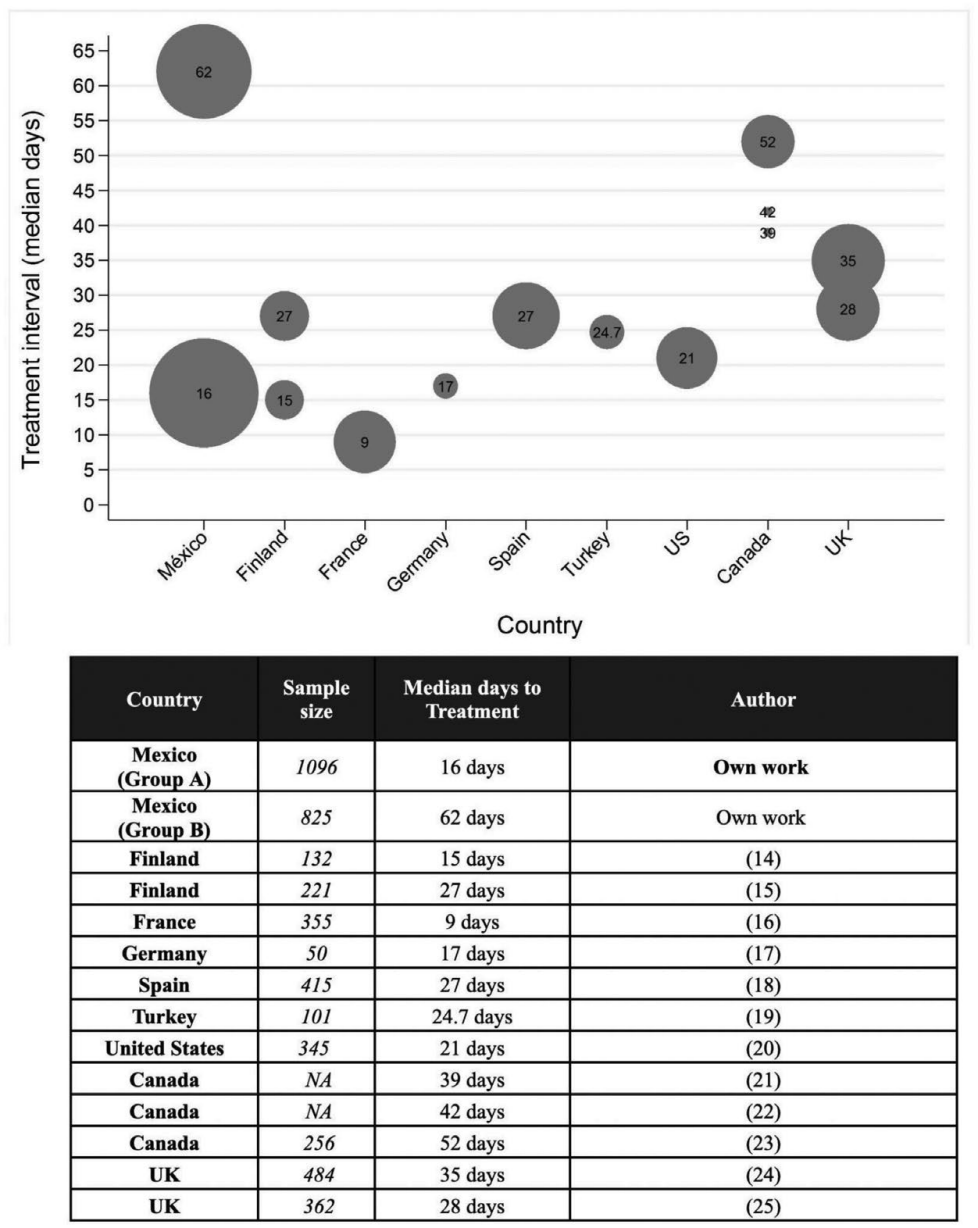
Comparison of treatment intervals and sample size against other studies in lung cancer at the international level.

A comparison of the median days to treatment for patients with lung cancer across the globe and their sample size is referenced in Figure 4. Compared to the international literature, in this study, both patient groups experienced treatment delays that were longer than those recommended by the NHS in the United Kingdom.^5^ This was particularly relevant for patients who underwent a diagnostic workup before arriving at INCAN, of which 80% had treatment delays of >31 days. More research needs to be done to understand why patients who are diagnosed with lung cancer externally and are admitted to the INCAN face more hurdles in their patient journey.

These findings highlight the social characteristics influencing treatment delays in patients with lung cancer in Mexico. In the literature, sex and age have been associated^16-21^ and not associated^22^ with more delays in the treatment interval for cancer. Similar to the results from this study, marital status has been associated with delays in the literature.^20,23,24^ Dyspnea is not highly predictive of lung cancer,^25^ hence patients with this symptom might encounter longer diagnostic intervals. However, the reasons for the increased likelihood of delays among this population in the treatment interval need to be studied further. Moreover, reduced delays in SCLC compared to NSCLC have also been previously evidenced in the literature.^9,16,26,27^ Unlike the literature,^7,21^ the sicker-quicker paradox does not stand in this population for this interval. However, these results show treatment differences play a role in delays in cancer care. Treatment delays across time might be due to differences in policies being implemented from 2000 onwards.^28-32^

In our sample, marital status, treatment type, year of diagnosis, first symptom at presentation, region of origin, and educational status seemed to influence the time to treatment initiation, albeit at different magnitudes depending on whether patients underwent their diagnostic workup before or after arriving at INCAN. Shedding light on the reasons why patient characteristics influence treatment delays can improve the efficiency of care delivery and help design strategies for reducing delays.

Reduced odds of delays in certain years, in either the internally or the externally diagnosed population, are representations of policies potentially taking place during particular periods of time.^28^ These periods are characterized by having different political parties involved in decision-making. More research is needed to understand the role of policies or interventions implemented across time and their impact in treatment intervals.

Furthermore, a noteworthy increase of approximately 1 week is visible in the treatment interval from 2014 onwards. This observation holds significance, given that each week of treatment delay is associated with a 3.2% reduction in survival for stage I NSCLC.^33^ In the advanced stages of the disease, it is anticipated that the impact of delays on survival will be even more pronounced.^33^ The increase in the time to treatment initiation from 2014 may be related both to an increase in lung cancer diagnoses and in referrals to INCAN, which strengthened and grew its lung cancer clinic during the studied period. Importantly, very few centers in Mexico have the capacity to provide multidisciplinary care for patients with cancer,^28,34^ and thus the increasing number of patients may slow the diagnostic and therapeutic process. Decentralizing care and improving access to cancer centers in other areas of the country is thus of the maximal importance in order to decrease the time to treatment initiation and to improve outcomes.^32,34,35^ Other efforts to streamline and organize care have shown promise in reducing delays. For example, some evidence suggests patient navigation initiatives may reduce the time to diagnosis and treatment, leading to greater patient satisfaction and in some instances better survival.^33,36-41^

Our study has some limitations. Our results are based on the population that is admitted to the INCAN, and may not be generalizable to the rest of the Mexican population. However, the sample is large and includes patients from different regions of the country. Since INCAN is the leading cancer center in Mexico,^34^ it is expected that patients treated in other cancer centers could experience worse delays. In addition, at the time of treatment some patients did not have a recorded diagnosis in their file, and although previous methods to reduce bias were used,^2^ these results might still be biased. As for this lost data, it could be that during the study period, some records were kept in paper format, while others were transferred to the electronic medical record. Alternatively, patients diagnosed externally could have experienced recall bias when referring their diagnosis date to the INCAN consultants. Finally, including the type of treatment could potentially lead to over adjustment, as the patients who experience certain stages of the disease are provided with particular treatment types. However, this study categorizes not only by type of treatment but also number of combined treatments.

## CONCLUSION

Our study sheds some light on the factors influencing treatment delays among lung cancer patients in Mexico, comparing those diagnosed internally at INCAN and those diagnosed externally. The findings reveal distinct outcomes for these patient groups, with external diagnoses associated with a higher likelihood of treatment delays beyond the recommended 31-day cutoff. Efforts to understand and address the complexities of patient journeys, considering factors like marital status, education, region, and first symptom at presentation, are crucial. Moreover, our study underlines the need for continued research to measure and improve cancer care delays in Mexico, with a particular focus on lung cancer.

In light of these findings, targeted interventions, such as improved referral systems, the use of information technology, and patient navigation initiatives, could play a pivotal role in reducing delays, and potentially improve the survival rates. As we move forward, it is imperative to validate and expand upon our results, fostering collaboration between healthcare professionals, researchers, and policymakers to optimize the efficiency of care delivery and improve outcomes for lung cancer patients in Mexico.

## Data Availability

The data underlying this article were provided by permission and through a collaboration agreement. Data will not be shared.
